# Active unilateral condylar hyperplasia: Assessment of the usefulness of single photon emission computed tomography

**DOI:** 10.4317/medoral.23699

**Published:** 2020-07-19

**Authors:** Vanesa Guerrero-Arenillas, David González-Padilla, Rosa Diaz-Sanchez, Daniel Torres-Lagares, José-Luis Gutiérrez-Pérez, Aida Gutiérrez-Corrales, María-Angeles Serrera-Figallo

**Affiliations:** 1 DDS. Collaborator. Oral Surgery Department, School of Dentistry, University of Seville; 2 DMD, OMFS, PhD. Oral and Maxillofacial Surgery Unit. University Hospital Virgen del Rocío of Seville; 3 DDS, PhD. Professor of Master in Oral Surgery. Oral Surgery Department, School of Dentistry, University of Seville; 4 DMD, OMFS, PhD. Oral and Maxillofacial Surgery Unit. University Hospital Virgen del Rocío of Seville. Professor of Oral Surgery. Oral Surgery Department, School of Dentistry, University of Seville

## Abstract

**Background:**

This study aims to evaluate whether the uptake difference by the condyles evaluated using single photon emission computed tomography (SPECT) examination is useful for predicting the activity of the feature and the advance of this pathology.

**Material and Methods:**

An observational and prospective study has been carried out on nine patients affected by unilateral condylar hyperplasia (UCH) with complete bone maturation, with a follow-up over 18 months. At the beginning of the study, a test-battery was conducted including dental casts, articular examination, teleradiography and cephalometry, computed tomography and SPECT, creating two groups of patients from a difference in uptake between both condyles greater than 10% over the follow-up period. Evolution of data obtained with the rest of the diagnostic tests were compared to confirm UCH activity predicted by SPECT.

**Results:**

The comparison of both groups did not show hardly any significant differences, with little clinical significance. Deviation of the mandibular line, the size of the branches or condyles behaved similarly in both study groups.

**Conclusions:**

From the data obtained in our study, we can conclude that the use of the difference in uptake between both condyles by applying the SPECT technique is not a valid approach for predicting clinical activity in cases of UCH.

** Key words:**Temporomandibular joint, facial asymmetry, single photon emission computed tomography, scintigraphy, condylar hyperplasia.

## Introduction

During the development of the facial skeleton, the mandibular condyles need to undergo similar and coordinated growth for facial symmetry and proper dental occlusion to occur. Said growth ends at the age of 15 years in women and at 18 years in men (1).

Condylar hyperplasia is a pathological condition characterised by abnormal and progressive growth of mandibular condylar cartilage (1). It is due to a non-neoplastic increase in the number of normal bone cells (1). It is infrequent, and in the majority of cases, it presents unilaterally and is associated with facial and mandibular asymmetry. It shows no preference for sex, race or altered laterality (1).

The aetiology of unilateral condylar hyperplasia (UCH) is uncertain and controversial, being considered a reactive process to a stimulus which is not known with any accuracy; however, we may assume that there are hormonal factors (somatomedin, growth factors such as IGF-1), as well as biomechanical (trauma), vascular, neoplastic (osteoma, osteochondroma and chondroma) or infectious which are involved in its development (2-4).

Clinical findings of UCH are facial and mandibular asymmetry, occlusal disorders with open or crossed bite, bone compensations and/or dental ones with overeruption and laterognathia, etc. Likewise, it is associated with episodes of pain, blockage or dislocation of the temporomandibular joint (TMJ) (1,5,6).

There are a multitude of diagnostic methods for detecting patients with condylar hyperplasia, the management of several of these methods being the instrument required for identifying the type of condylar hyperplasia and the facial or occlusal change present in the patient (7-9).

Although the signs and symptoms may lead to a correct diagnosis, help needs to be sought using imaging and cephalometric measurements to obtain more information, since the patient may remain in an active phase, with mandibular condylar growth, or in an inactive or stationary phase, where condylar growth is halted (1-7).

Besides cephalometric methods for evaluating facial asymmetry, Single Photon Emission Computed Tomography (SPECT) has been the diagnostic method chosen up to now for the definitive diagnosis of UCH activity. SPECT is an imaging method based on the injection of phosphates marked with radionuclides providing a functional image which shows the metabolic response of the object being evaluated. The amount of trace material is controlled by the level of metabolic activity and/or blood supply in the region emitting radiation which is detected by gamma cameras. SPECT is able to enable three-dimensional reconstructions, it combines a graphic and numerical representation of the pixel count, and is an excellent means for quantifying and comparing osteoblast activity in condyles and drawing up a suiTable treatment plan. A variant of the technique is SPECT/CT where it is associated with the implementation of Computed Tomography for measuring structures of interest (7-19).

Until now, SPECT has been the diagnostic method of choice for confirming UCH activity. For confirming said activity, a difference of more than 10% uptake between condyles has been taken as a reference (14). However, in the light of various publications taken from the literature (16,20), it is not clear that the use of SPECT is the ideal radiological method for establishing said diagnosis and a certain degree of controversy is maintained in this statement.

This point within UCH management is essential, because once the diagnosis of UCH in a patient has been made, what the development of condylar and mandibular growth will be is unknown and, consequently, how it will affect the facial and occlusal appearance of the patient over time (20). It is for this reason that diagnosis in patients with active UCH would enable its clinical development to be foreseen, and it would enable the possibility of implementing early treatment thus avoiding the emergence of more serious deformities over time.

The study aims to determine the reliability of the use of Single Photon Emission Computed Tomography (SPECT) in the diagnosis of unilateral condylar hyperplasia activity focusing on the correct forecast of the possible development and worsening of the asymmetry diagnosed.

The study hypothesis is that, within the population diagnosed with UCH, the existence of an uptake difference greater than 10% between both condyles corresponds to active hyperplasia which must be endorsed clinically within a prudential period which in this study has been held at 18 months.

The hypothesis is important because many clinical decisions are taken bearing in mind or employing this concept of active hyperplasia based on this test (13).

## Material and Methods

- Study design

The design we have used for implementing the SPECT analysis as a diagnostic method of unilateral condylar hyperplasia activity has been an observational, analytic and prospective study on nine patients suffering from unilateral condylar hyperplasia with complete bone maturation (in accordance with the extended Björk stages) (21).

This study was approved by the Ethics Committee of the Virgen del Rocío University Hospital, undertaken between January 2011 and December 2016 and has been financed by the Andalusian Regional Department of Health.

Before participation, the purpose and procedures were explained in detail to all the volunteers, and all the participants gave their informed consent in writing in accordance with the Helsinki declaration. The study was designed, implemented, analysed and reported in accordance with the “Good Clinical Practice” standards.

- Inclusion and exclusion criteria

The inclusion criteria for patients in this study have been: patients with unilateral condylar hyperplasia. Diagnosis was carried out based on the following clinical and x-ray criteria: patients with clear facial and mandibular asymmetry identified by two maxillofacial surgeons, together with differences in shape and volume between both condyles, confirmed using a CT scan (22); patient, or in the case of an under-age patient, father, mother or guardian who has been informed by the researcher of the aims, risks and benefits of the study, as well as the corresponding obligations and whose consent for participating in the study has been given in writing; patient, or in the case of an under-age patient, father mother or guardian who understands the need for cooperating throughout the duration of the study.

Exclusion criteria for patients in this study have been: Patient without unilateral condylar hyperplasia; patient with a history of trauma, infection or neoplasia in any temporomandibular joint; patient with general bone syndrome; patient with x-ray signs characteristic of another condylar pathology; patient treated previously with dental facial orthopaedic or orthognathic surgery; patient with a physical, mental, psychological or linguistic pathology which may limit the understanding or adherence to the study.

- Study development and evaluation of the participants

All patients included in the study underwent an exhaustive examination at the beginning of the study and 18 months later, in an attempt to identify any change in the growth of the structures studied. Said tests were as follows.

Plaster casts of the upper and lower arcade and bite registration for analysis of deviation of the dental midline (mm) were taken. A detailed clinical examination of the TMJ in which ranges of mandibular mobility (maximum opening, laterality and protrusion) were observed for which we used Therabite® motion scales (Atos Medical Spain SL, Spain) and expressed measurements in mm.

Likewise, subjective pain was present in the various muscle groups (temporal, masseter, internal pterygoid, lateral pterygoid and sternocleidomastoid) and the left and right TMJ itself was examined. The existence of opening and closing snaps in both TMJs.

Lateral and frontal teleradiography of the cranium was carried out using an analogue Strato 2000 Panoramic X-Ray unit by Villa Sistemi Medicali S.P.A (Milan, Italy). It was carried out at a focal distance from the plate of 1.5m., Penetration (Kv) range between 60 and 90 KV and the intensity of the x-rays was between 6 and 15mA., parameters which are regulated depending on the size of the cranium to be studied. Likewise, the apparatus consisted of a system of filters for the soft parts of the anterior side of the face (nose, lips and chin on the output tube of the x-ray device in order to mitigate the amount of radiation they receive and to be able to see them together with the harder tissues (bone structures) on the plate itself).

After obtaining the x-ray tests indicated above, various cephalometric analyses were carried out to determine UCH using orthodontic measurement parameters.

Firstly, Ricketts frontal cephalometric analysis was carried out. Ricketts frontal cephalometric analysis is currently used in the American Institute for Bioprogressive Education, it is based on classic frontal cephalometric analysis, although with a different order, and it includes some new measurements. The same structures of the classic analysis must be represented and besides the first lower premolars and the mid-palatal suture: the points and planes are the same. This study includes two fields of study, the skeletal problem and the dental problem (6).

Secondly, a Jarabak lateral cephalometric analysis was performed. Jarabak analysis is useful to determine qualitative and quantitative growth characteristics, that is, growth direction and potential. Furthermore, it contributes to a better definition of facial biotypology. Jarabak’s polygon is efficient at detecting the reaction they will have compared to therapeutic procedures for those patients belonging to not very well-defined biotypes. For Jarabak, the basis of diagnosis is to build up the areas of overlap essential for case planning and its subsequent evaluation, enabling better vision of the case to be obtained with the fewest cephalometric measurements (6).

Both Ricketts frontal cephalometry and Jarabak lateral cephalometry were performed using Dolphin software (Dolphin Imaging and Management Solutions, California, USA).

A SPECT-CAT scan was carried out in DICOM format. To study the three-dimensional SPECT-CAT tomographic x-ray images, we used VirSSPA software (12), a virtual reality program which generates 3D models developed by the Andalusian Regional Government (Spain). The presence or absence of unilateral condylar hyperplasia was evaluated using a volumetric bilateral condylar and bilateral mandibular study (9). Selecting the cranial bone tissue using the seed segmentation method, we analysed the following parameters: Right and left linear mandibular condyle length (cm), right and left linear ascending branch mandibular length (cm), right and left surface mandibular hemibody length (cm), right and left linear mandibular hemibody length (cm), right and left mandibular condyle volume (cc), right and left ascending branch mandibular volume (cc) and right and left mandibular hemibody volume (cc).

Finally, a SPECT scintigraphy of bilateral TMJ was carried out in two stages and a Selective Bone SPECT-CT with an analysis of uptake percentage between mandibular condyles using mean region of interest (ROI). The radiopharmaceutical used was Tc99m in the form of hydroxymethylene diphosphonate (HDP) at an intravenous dose of 22 mCi. Instrumentation was implemented using a Siemens Symbia T6 gamma camera with a low energy and high-resolution collimator (Siemens AG, Germany). The estimated effective dose was 4.1 mSv for an administered dose of 740 MBg. The uptake differential between the right condyle and the left condyle of the TMJ was analysed to be able to examine in this way the presence of active unilateral condylar overgrowth and to be able to establish, using this parameter, the study groups to carry out the study in question.

Two groups of studies were determined depending on the uptake difference between both condyles in accordance with the criteria delivered by Saridin *et al*. (14), establishing as a study group those patients with an uptake difference between both condyles greater than 10% maintained for the 18-month follow-up, and as the control group, those patients with a difference below 10% between uptake between condyles. After taking this reference, we established our study groups, five patients were selected for the experimental group compared to four patients for the control group.

- Statistics

Statistically, final data were taken and they were compared with the initial ones from the diagnostic tests carried out, giving rise to values which summarise the development of both groups over the 18-month follow-up period. The qualitative variables (Yes/No) were quantified by coding as a 0 value the No value and with the 1 value the Yes value, in order to make the comparison of said variables easier. Said values were compared using a non-parametric test (Mann-Whitney U test). A *p*<0.05 value was assumed to be significant.

## Results

The average age at the start of the study was 19.89 ± 7.76 years. There was a total of four men compared to five women in the participants in this study. Data obtained from the SPECT determined that five participants belonged to the study group (uptake difference greater than 10% between condyles) compared to four in the control group (uptake difference less than 10% between condyles) (Table 1).

Regarding the data, we can see in Table 2, both the data relating to the displacement of the lower midline and those relating to the joint examination did not show significant differences between both groups. What is more, in fact, in some variables, such as displacement of the lower midline, it is greater in the control group than in the experimental one.

In respect of the measurements obtained from the cephalometric measurements on Ricketts frontal cephalometry, we can focus on the maxillo-mandibular midline, (Maxillo-Mand Midline (mm)), as the most useful data in the control of these patients because it analyses the deviation of the chin compared to the upper maxilla. The data provided by this measurement in the development of both groups is not statistically significant. What is more, there is greater variation in the control group than in the experimental one.

In the Jarabak lateral cephalometry one of the most important measurements is the height of the ascending branch (Ramus Height (Ar-Go) (mm)) and the posterior face height ((Posterior Face Height (SGo) (mm)). In both measurements, the behaviour of both groups is similar, of course, without reaching statistically significant differences between them (Table 2).

Finally, the measurements on length of the mandibular branch or the condylar volume using CBCT in the initial and final stages of the study in patients in both groups, neither variations nor different behaviours were identified between both groups of patients (Table 2).

**Table 1:**
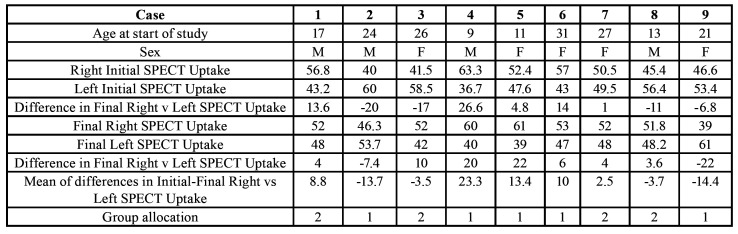
Characteristics of the patients being studied, uptake per condyle at the start of the study and at 18-month follow-up, differences between both initial and final uptakes and difference of average uptake between both condyles during the 18-month follow-up, which gave rise to the allocation to the study group (> than 10%) or control group (< than 10%) (Group 1 – Study / Group 2 – Control).

**Table 2:**
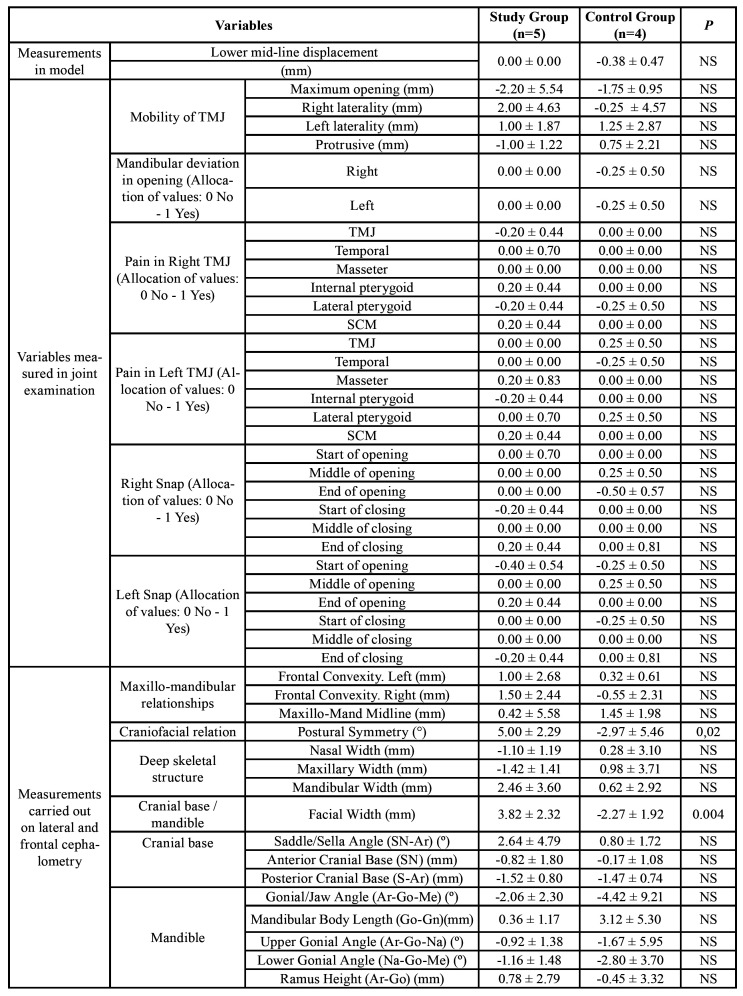
Change in controlled variables during the 18-month follow-up period of the study (Postural symmetry, the only measurement for the craniofacial relation, is the difference in angles (left and right) formed by a plane from the zygomatic suture to the antegonion and the antegonion to the zygomatic arch).

**Table 2 cont.**
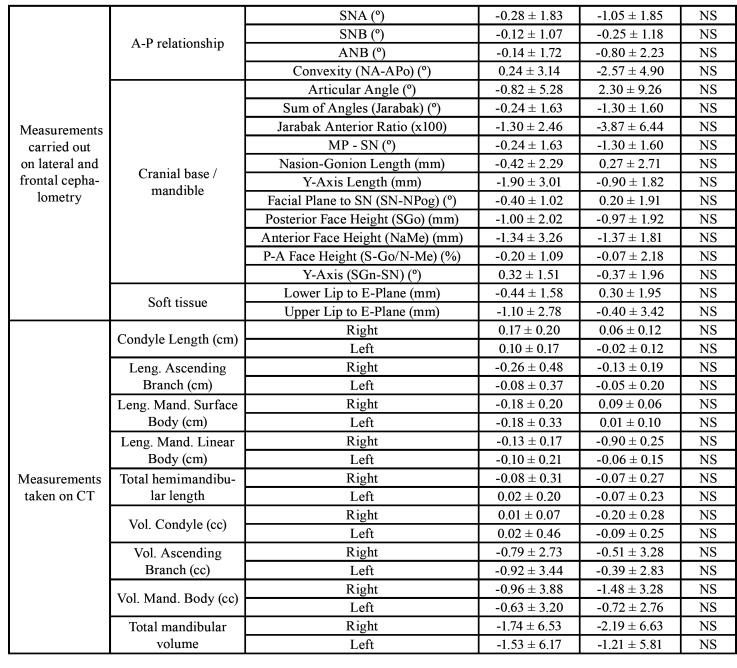
Change in controlled variables during the 18-month follow-up period of the study (Postural symmetry, the only measurement for the craniofacial relation, is the difference in angles (left and right) formed by a plane from the zygomatic suture to the antegonion and the antegonion to the zygomatic arch).

## Discussion

According to the published literature, a difference in scintigraphic uptake between condyles greater than 10% is a pathognomonic data for the detection of UCH activity, and this is the value we have used to determine our distribution of subjects in the study group (supposedly affected by active UCH) and the control group (supposedly with no activity) (7). Saridin *et al*. studied SPECT findings in patients with condylar hyperplasia with and without progression of facial asymmetry (n=26 in each group) and concluded that the best data for identifying each group of patients was the comparison of condylar hyperactivity between both condyles (13). Likewise, they carried out a meta-analysis on the published literature concluding that condylar uptake difference considered normal is less than 10% (14).

Various authors have corroborated setting condylar uptake difference at 10% (15,17,23,24). However, Kajan *et al*. studied 38 patients, with an age range between 13 and 34 years, using SPECT, giving a lower percentage in condylar uptake difference of healthy patients and those affected with UCH, concluding that the maximum variation in uptake between both healthy condyles in a patient without mandibular hyperplasia is 6.2% (12).

However, the data obtained in our study shows how this difference in uptake does not ensure the active development of the disease, even in patients with complete bone maturation in accordance with Björk’s stages. At the beginning of the study, we performed this test to establish two groups according to UCH activity and we established as the study group those patients with scintigraphic uptake difference greater than 10%. However, after an 18-month follow-up of the study group patients there has been no evolution in their development of UCH, there being no statistically significant differences in any of the parameters studied, consequently we can state that these results suggest that stability in facial asymmetry in patients with complete bone maturation is not related to uptake difference between both condyles.

Robinson *et al*. published a study in 1990 with similar results to ours with another type of scintigraphy to carry out the diagnosis, planar scintigraphy, since they detected persistence in condylar uptake in the study in 6 of 10 patients with clinically inactive UCH (19). Henderson *et al*. obtained similar results in their study of 14 patients with UCH (18). These authors found that an increased unilateral uptake of the radioisotope in planar scintigraphy could often indicate abnormal active condylar growth. However, they determined that the false positives could be found in patients with TMJ diseases giving altered results in scintigraphy (18,25).

After these results, many authors published that the diagnosis made using SPECT replaced to a greater extent planar scintigraphy in the evaluation of growth in mandibular asymmetry due to the fact that it is a more reliable technology and without limitations in the positioning of the patient or overlapping with other bone structures, finding a condyle uptake percentage related to the 55% indicative of activity (11). Chan *et al*. compared planar scintigraphy with SPECT in 23 patients with UCH and 16 healthy subjects, finding an uptake difference between normal condyles of 7% in SPECT and 5% in planar scintigraphy (10), and determining that the use of SPECT was the technology of choice in the diagnosis of UCH activity and being corroborated by various authors (26-30), who furthermore concluded that the sensitivity of SPECT is between 88 and 95% and specificity between 61.1 and 77% (29,30).

However, as we have mentioned earlier, the fact that there is a condylar uptake difference greater than 10% does not imply the development and advance of the disease. To do so, we have compared data with orthodontic parameters during the 18 months of the study in each individual, and we have compared the data obtained, not finding any statistically significant difference between both temporal points. Hodder *et al*. in the year 2000 studied 18 patients with UCH and 11 control subjects and although they considered abnormal a relative uptake percentage in SPECT of the condyle affected greater or equal to 55%, they added that the diagnosis of condylar activity must be associated with a clinical, cephalometric and x-ray evaluation of the patient for therapeutic decision making, since in all cases there was no development or advance in UCH (16). The results obtained in our study and others such as that of Hodder *et al*. may be as such because SPECT results are only a snapshot of the relative growth rate between condyles at a given time as Karssemakers *et al*. stated (16,20). These authors concluded that the diagnosis of UCH activity should be based on the combined use of SPECT and the clinical history of the patient in order to avoid erroneous diagnoses resulting from obtaining results at a specific time of a dynamic process such as growth (20).

## Conclusions

After implementing this study, we can conclude that the differential percentage in uptake of the mandibular condyles in scintigraphy is not a valid diagnostic method for predicting the evolution of patients with unilateral condylar hyperplasia. The use of CT scans and the taking of measurements of mandibular condyle volume, mandibular ascending branch, mandibular body and total hemimandible are valid diagnostic data in the detection and control of the evolution of patients with unilateral condylar hyperplasia.
